# Disproportionate mitral regurgitation: another myth? A critical appraisal of echocardiographic assessment of functional mitral regurgitation

**DOI:** 10.1007/s10554-020-01975-6

**Published:** 2020-08-26

**Authors:** Andreas Hagendorff, Fabian Knebel, Andreas Helfen, Stephan Stöbe, Torsten Doenst, Volkmar Falk

**Affiliations:** 1 Department of Cardiology, University of Leipzig, Leipzig, Germany; 2 Department of Cardiology, University of Berlin, Charité-Universitätsmedizin Berlin, Berlin, Germany; 3 Speaker of the Working Group „Cardiovascular Ultrasound“ of the German Society of Cardiology, Düsseldorf, Germany; 4 Department of Cardiology, St. Marien Hospital Lünen, Lünen, Germany; 5 Co-Speaker of the Working Group „Cardiovascular Ultrasound“ of the German Society of Cardiology, Düsseldorf, Germany; 6 Department of Cardiothoracic Surgery, Jena University Hospital, Friedrich Schiller University of Jena, Jena, Germany; 7 Department of Cardiothoracic and Vascular Surgery, University of Berlin, Charité-Universitätsmedizin Berlin, Berlin, Germany; 8 Department of Cardiothoracic and Vascular Surgery, German Heart Center Berlin, Berlin, Germany; 9 Department of Health Science and Technology, Swiss Federal Institute of Technology, Zurich, Switzerland; 10 German Center of Cardiovascular Research, Partner Site Berlin, Berlin, Germany; 11 Department of Cardiology, University Hospital Leipzig, Liebigstrasse 20, 04103 Leipzig, Germany

**Keywords:** Functional mitral regurgitation, Disproportionate mitral regurgitation, Hemodynamics, Regurgitant fraction, Echocardiography

## Abstract

The contradictory findings of recent prospective randomized controlled trials assessing the impact of percutaneous edge-to-edge repair in patients with functional or secondary mitral regurgitation have triggered a lively discussion about an “integrated” echocardiographic approach for grading severity of mitral regurgitation. In the MITRA-FR trial, the COAPT trial and the REDUCE-FMR trial echocardiographic assessment of the severity of mitral regurgitation was consistent with principles set forth by the current echocardiographic guidelines and analysed in its best settings by expert international leaders in the field of echocardiography. However, serious inconsistencies appeared in the presented echocardiographic assessments regarding cardiac output and regurgitant fraction. A new term “disproportionate functional mitral regurgitation” was introduced describing a situation where the increase of effective regurgitant orifice area exceeds the enlargement of the left ventricular end-diastolic volumes. Further discussion resulted in the idea of a “new conceptional framework” for distinguishing “proportionate” and “disproportionate” functional mitral regurgitation. The aim of this viewpoint is to dispute conclusions based on the term “disproportionate” mitral regurgitation. A “disproportionate” FMR is highly questionable because disproportionateness of flow in communication vessels cannot exist. In addition, a proposal of echocardiographic assessment based on a conventional comprehensive transthoracic echocardiography is given to avoid obvious hemodynamic contradictions.

## Background

Inconsistencies between the left ventricular (LV) total stroke volume (LVSV_tot_) obtained with two-dimensional (2D) planimetry by transthoracic echocardiography (TTE), the Doppler-derived effective LV forward SV (LVSV_eff_) and the mitral regurgitant volume (RegVol_MV_) obtained with the 2D proximal isovelocity surface area (2D-PISA)-method can be observed in all recent transcatheter mitral valve repair (TMVR) trials [1–3]. Taken together, the mean values of the echocardiographic parameters presented in these trials characterize a hemodynamic state resembling conditions below cardiogenic shock index or inconsistent with severe functional mitral regurgitation, FMR [4–7]. The so-called “integrated approach” of grading FMR severity can be scrutinised because the characterization of the hemodynamics by echocardiography in patients with FMR obviously had failed [8, 9]. Thus, a proposal like „a specific integrative multiparametric MR grading algorithm that could identify a homogeneous population that would benefit from TMVR“ [10] is in doubt. This viewpoint might contribute to the scientific debate for the need of more conclusive echocardiographic FMR assessment as applied in the recent TMVR trials. In light of the fact, that the echocardiography performed in the TMVR trials is presumably divisions above whatever is done in routine practice, and data analysis was performed by international expert leaders, the attempts to explain the differences of inconsistent echocardiographic data [1–7, 10–23] have to be critically discussed. If we still want to use echocardiography to assess FMR, because it is the most common tool, which can be used, we need substantial methodological improvements.

The first objective of this viewpoint is to discuss whether a “disproportionate” FMR can be possible anyway with the conclusion that disproportionateness of flow in communication vessels cannot exist. The second objective is to propose an extended transparent echocardiographic protocol focusing on hemodynamic plausibility to improve the grading FMR severity.

## General rheological considerations in valvular heart diseases

**Fig. 1:**
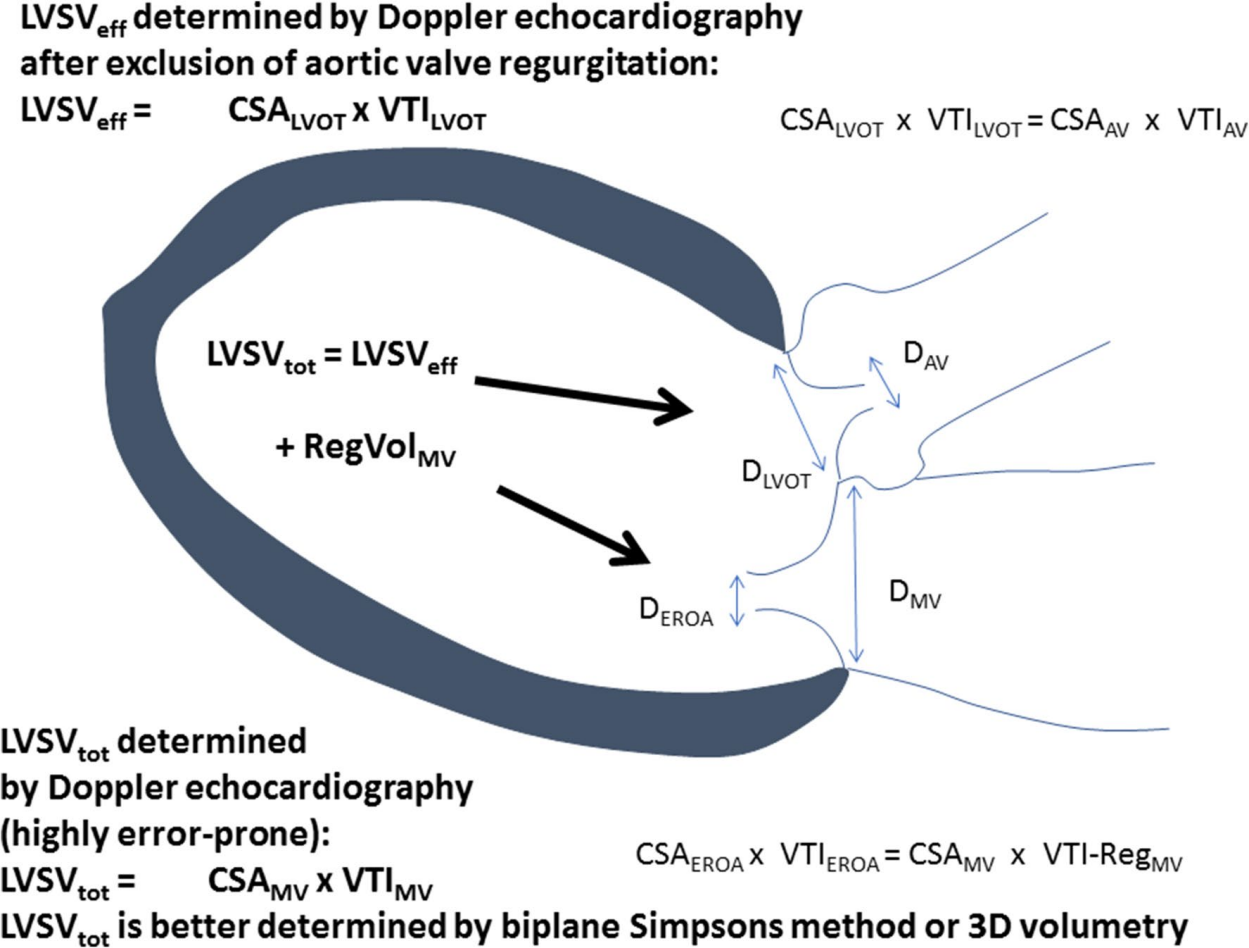
Illustration of the proportionality between cross sectional areas and forward and backward systolic blood flow in the left ventricle as a system of communicating tubes. Transmitral regurgitant blood flow volume is proportional to the regurgitant orifice area, if a single timepoint measurement is performed by echocardiography. *CSA*_*AV*_ cross section area of the aortic valve orifice, *CSA*_*EROA*_ cross section area of the mitral valve regurgitant orifice, *CSA*_*LVOT*_ cross section area of the left ventricular outflow tract, *CSA*_*MV*_ cross section area at the level of the mitral valve annulus, *D*_*AV*_ diameter of the aortic valve orifice, *D*_*EROA*_ diameter of the mitral valve regurgitant orifice, *D*_*LVOT*_ diameter of the left ventricular outflow tract, *D*_*MV*_ diameter at the level of the mitral valve annulus, *LVSV*_*eff*_ left ventricular effective stroke volume, *LVSV*_*tot*_ left ventricular total stroke volume, *RegVol*_*MV*_ transmitral regurgitant volume, *VTI*_*AV*_ velocity time integral of the systolic forward blood flow through the aortic valve orifice, *VTI*_*EROA*_ velocity time integral of the diastolic backward blood flow through the mitral valve regurgitant orifice, *VTI*_*LVOT*_ velocity time integral of the systolic forward blood flow through the left ventricular outflow tract, *VTI*_*MV*_ velocity time integral of the diastolic forward mitral flow at the level of the mitral annulus, *VTI-Reg*_*MV*_ velocity time integral of the systolic regurgitant transmitral blood flow at the level of the mitral valve annulus

The calculation of the effective orifice area by the continuity equation is an accepted method for echocardiographic grading of aortic valve (AV) severity based on the physical laws of conservation of mass and energy. These principles cannot be neglected meaning that blood flow velocities at defined orifices are proportional in a system of communicating tubes. If we assume “pure” aortic valve stenosis (AS), and if cross sectional areas are known at the level of the left ventricular outflow tract (LVOT) as well as at the level of the stenotic orifice area, LVSV_tot_ can be measured at the level of the LVOT as well as at the level of the stenotic orifice area by Doppler echocardiography, because volume flow has to be the same at both levels [24, 25]. In “pure” mitral regurgitation the same principle can be applied at the regurgitant orifice area; RegVol_MV_ is the calculated difference LVSV_tot_ − LVSV_eff_. Thus, if forward stroke volume LVSV_eff_ is known, the interrelationship between effective regurgitant orifice area (EROA) and regurgitant flow can be calculated due to physical laws of rheology (Fig. 1).

**Fig. 2:**
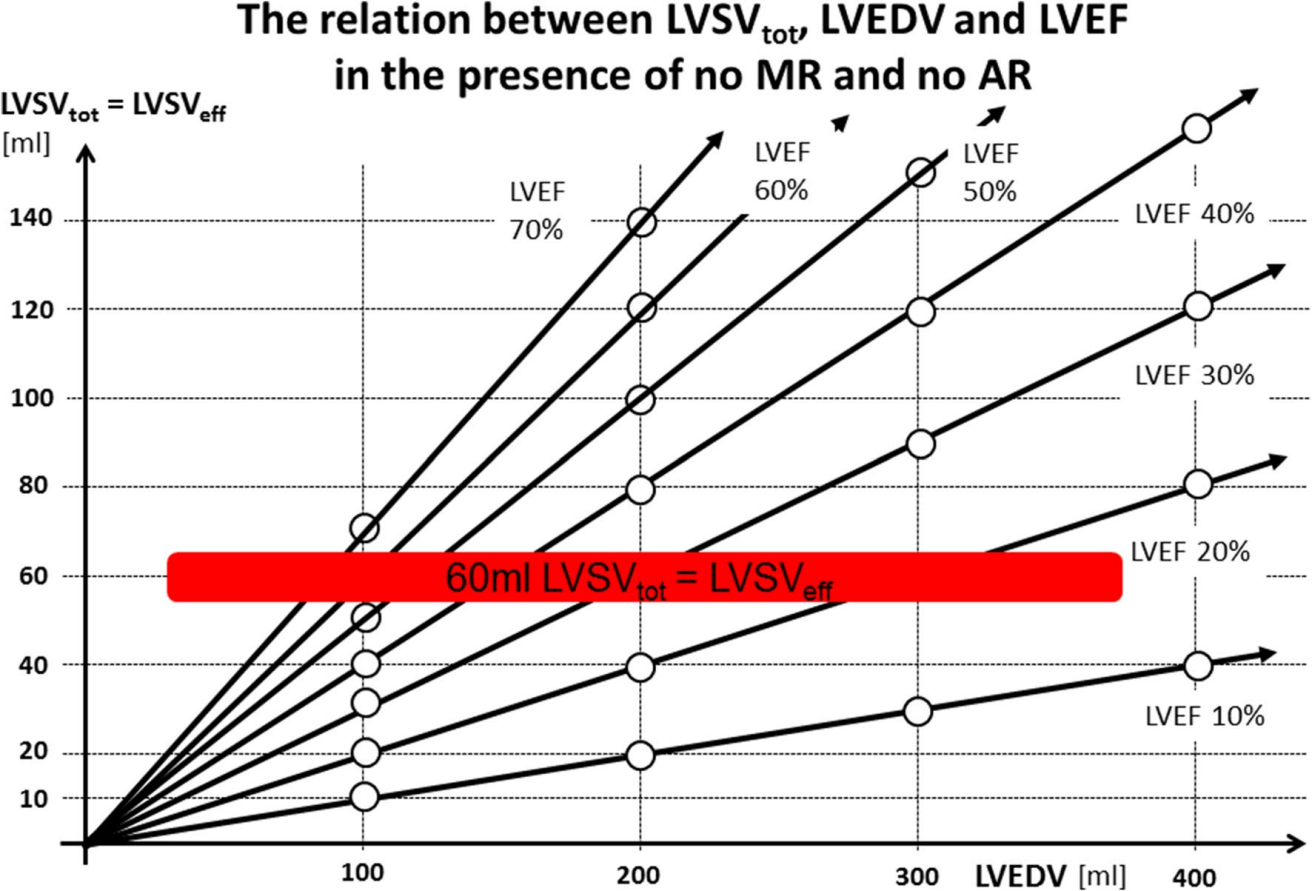
The relation between total left ventricular (LV) stroke volume (LVSV_tot_) and LV end-diastolic volume (LVEDV) with respect to LV ejection fraction (LVEF) in the presence of no mitral regurgitation (MR) and no aortic regurgitation (AR): *LVSV*_*eff*_ LV effective stroke volume, *CO* cardiac output, *CI* cardiac index (additional explanations in the text)

To introduce into the hemodynamic discussion in FMR the parameters cardiac output (CO) and the cardiac index (CI) determined by the LVSV_eff_, heart rate (HR) and body surface area (BSA) should be the basis of the physiological thinking in echocardiography. Further important cardiac parameters are the LV end-diastolic volume (LVEDV) and the LV ejection fraction (LVEF) to interpret the cardiovascular physiology. Normal CO is defined within ranges of 4.0 to 4.5 l/min or a CI of about 2.5 l/min/m^2^. A CI < 2.2 l/min/m^2^ is a criterium for cardiogenic shock (CO < about 4 l/min). A normal heart rate at rest—and the target heart rate for cardiovascular patients with optimal medical treatment (OMT)—is within the ranges of 50 to 70/min. With respect to these values of a normal cardiovascular physiology a borderline region to differentiate between normal conditions and cardiac decompensation can be marked in a LVSV_eff_ − LVEDV diagram with respect to different LVEF (Fig. 2). To ensure sufficient cardiac output it is obvious that no mitral as well as aortic regurgitation is present, because LVSV_tot_ must be equal to LVSV_eff_ (Fig. 2).

**Fig. 3:**
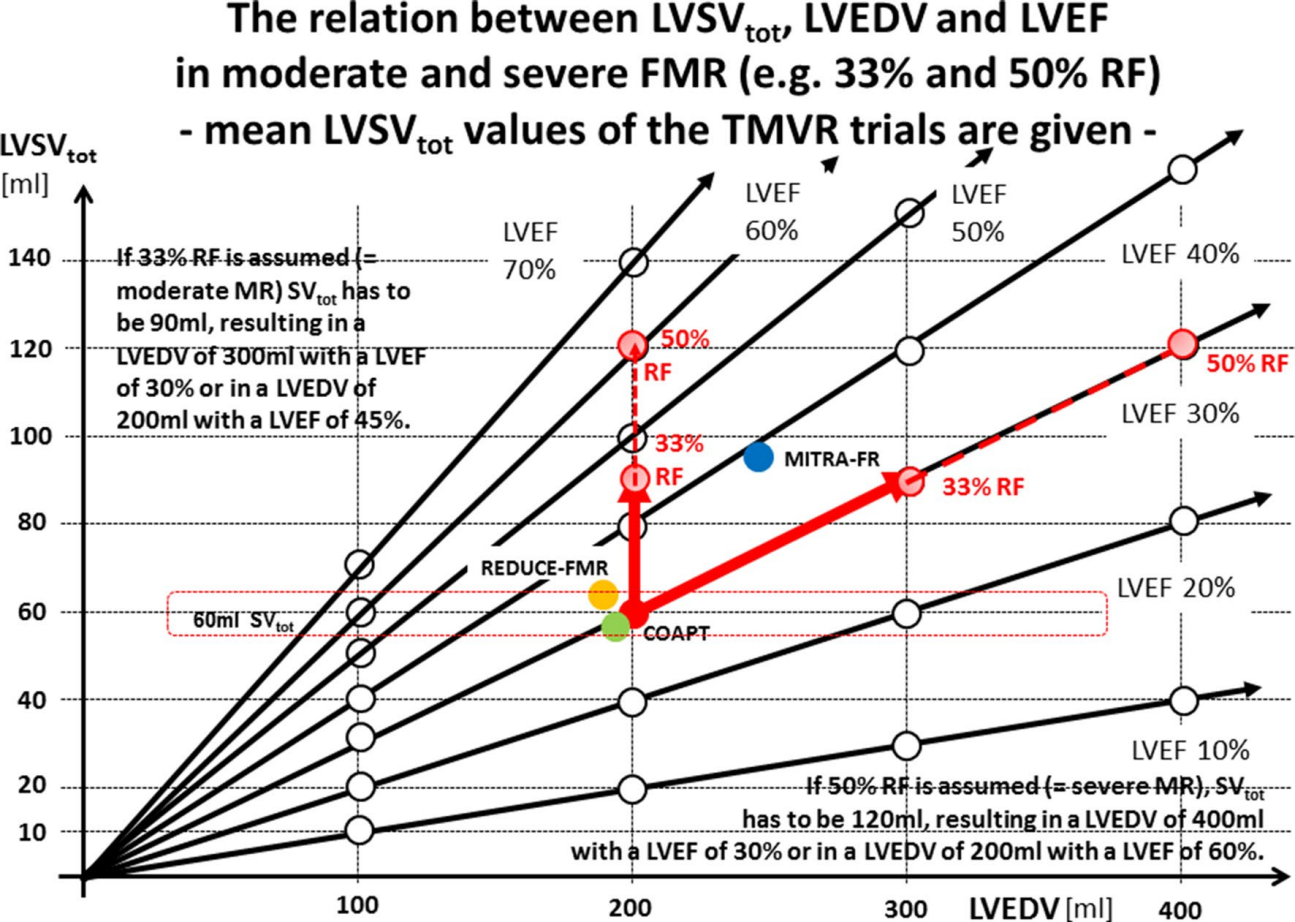
The relation between total left ventricular (LV) stroke volume (LVSV_tot_), LV end-diastolic volume (LVEDV) and LV ejection fraction (LVEF) in moderate and severe mitral regurgitation (MR) (e.g. 33% and 50% RF). *LVSV*_*eff*_ LV effective stroke volume, *CO* cardiac output, *CI* cardiac index. Mean values of LVSV_tot_ and LVEDV presented in recent transcatheter mitral valve repair (TMVR) trials are shown in colored dots (green dots COAPT, blue dots MITRA-FR, and orange dots REDUCE-FMR). The red dot displays a left ventricle with a LVEDV of 200 ml and a LVSV_eff_ of 60 ml; the calculated LVSV_tot_ and LVEDV values in the presence of a LVEF of 30% in a moderate FMR (regurgitant fraction, RF = 33%) are LVSV_tot_ = 90 ml and LVEDV = 300 ml (hollow red dots), in a severe FMR (RF = 50%) are LVSV_tot_ = 120 ml and LVEDV = 400 ml (hollow red dots); the calculated LVSV_tot_ and LVEF values in the presence of LVEDV of 200 ml in a moderate FMR (RF = 33%) LVSV_tot_ has to be 90 ml and LVEF 45% (hollow red dots), in a severe FMR (RF = 50%) LVSV_tot_ has to be 120 ml and LVEF 60% (hollow red dots) (additional explanations in the text)

If the mean values of LVSV_tot_ and LVEDV of the recent TMVR trials [1–3] are put into the diagram of Fig. 2, the colored dots represent the respective relationships (Fig. 3). Table 1 illustrates the hemodynamic parameters of the recent TMVR trials reported in the literature as well as the assumptions resulting from logical calculations of the presented data [1–3]. To explain the differences in the COAPT trial version 1 is corresponding to the presented data of the original paper [2], version 2 is corresponding to the authors reply to the letter to the editor [4]. In this reply the authors issued the statement “The actual mean forward stroke volume in the COAPT trial as measured with Doppler was 51 ml, and the regurgitant volume as measured with the use of the PISA method was 59 ml, values that are consistent with severe mitral regurgitation” [4, 17, 18]. However, if the forward stroke volume is subtracted from the LVSV_tot_, the RegVol_MV_ is only 8 ml, revealing a difference of 51 ml RegVol_MV_ (RegVol_MV_ = LVSV_tot_ − LVSV_eff_) or a difference of 51 ml LVSV_tot_ (LVSV_tot_ = LVSV_eff_ + RegVol_MV_) in the device group, which is not explained by the COAPT authors (Table 1).

**Table 1:** Summary of mean values ± SD reported in COAPT, MITRA-FR and REDUCE-FMR and *calculated values based on the reported mean values*

Characteristics	MITRA-FR		COAPT (version 1)		COAPT (Version 2)		REDUCE-FMR	
	Device group	Control group	Device group	Control group	Device group	Control group	Device group	Control group
LVEDV	136.2 ± 37.4 ml/m^2^	134.5 ± 33.1 ml/m^2^	194.4 ± 69.2 ml	191.0 ± 72.9 ml	194.4 ± 69.2 ml	191.0 ± 72.9 ml	187.0 ± 65.6 ml	188.6 ± 75.7 ml
	Assumption: BSA 1.8 m^2^	Assumption: BSA 1.8 m^2^						
	245.2 ± 67.3 ml	242.1 ± 59.6 ml						
LVESV calculation: LVEDV − LVSV_eff_	245 − 82 = Mean: 163 ml	242 − 80 = Mean: 162 ml	135.5 ± 56.1 ml	134.3 ± 60.3 ml	135.5 ± 56.1 ml	134.3 ± 60.3 ml	127.4 ± 56.1 ml	122.0 ± 59.8 ml
LVSV_tot_ (= LVEDV − LVESV)			Mean: 59.4 ml	Mean: 56.7 ml	Mean: 59.4 ml	Mean: 56.7 ml	Mean: 59.6 ml	Mean: 66.6 ml
Calculation: LVEDV/100 × LVEF	Calculated from LVEDV and LVEF: mean: 82 ml	Calculated from LVEDV and LVEF: mean: 80 ml					Calculated from LVEDV and LVEF: mean: 64 ml	Calculated from LVEDV and LVEF: mean: 70 ml
LVSV_eff_ (= LVSV_tot_ − RegVol_MV_)	82 − 45 = Mean: 37 ml	80 − 45 = Mean: 40 ml	< 6.4 ml	< 4.7 ml	< 6.4 ml	< 4.7 ml	64 − 40 = 24 ml	70 − 38 = 32 ml
LVSV_eff_ (= LVSV_Dopp_)	–	–	–	–	51	51	–	–
LVEF (%)	33.3 ± 6.5	32.9 ± 6.7	31.3 ± 9.1	31.3 ± 9.6	31.3 ± 9.1	31.3 ± 9.6	34 ± 9	37 ± 9
HR	73 ± 13 min^−1^	72 ± 13 min^−1^	Assumption: 65–70 min^−1^	Assumption 65–70 min^−1^	Assumption: 65–70 min^−1^	Assumption 65–70 min^−1^	70 ± 13 min^−1^	70 ± 11 min^−1^
CO	Mean: 2.70/min	Mean: 2.88/min	Mean: 0.42–0.45 l/min	Mean: 0.31–0.33 l/min	Mean: 3.32–3.36 l/min	Mean: 3.32–3.36 l/min	Mean: 1.68 l/min	Mean: 2.24 l/min
CI	Assumption: BSA 1.8 m^2^	Assumption: BSA 1.8 m^2^	Assumption: BSA 1.8 m^2^	Assumption: BSA 1.8 m^2^	Assumption: BSA 1.8 m^2^	Assumption: BSA 1.8 m^2^	Assumption: BSA 1.8 m^2^	Assumption: BSA 1.8 m^2^
	1.50 l/min/m^2^	1.60 l/min/m^2^	0.23–0.25 l/min/m^2^	0.17–0.18 l/min/m^2^	1.84–1.87 l/min/m^2^	1.84–1.87 l/min/m^2^	0.93 l/min/m^2^	1.24 l/min/m^2^
RegVol_MV_ (= LVSV_tot_ − LVSV_eff_)	82 − 37 = 45 ml → RF = 44.9% → MR 4 +	80 − 40 = 40 ml → RF = 50.0% → MR 4 +	MR 3 + 49%	MR 3 + 55%	59.4 − 51 = → 8.4 ml → RF = 14.1% → MR 0–1	56.7 − 51 = 5.7 ml → RF = 10.1% → MR 0–1	64 − 24 = 40 ml → RF = 62.5% → MR 4 +	70 − 32 = 38 ml → RF = 54.3% → MR 4 +
			MR 4 + 51% → > 45–60 ml	MR 4 + 45% → > 45–60 ml				
			Minimum mean value: 53 ml	Minimum mean value: 52 ml				
RegVol_MV_ (2D-PISA)	45 ± 13 ml	45 ± 14 ml	59 ml	59 ml	59 ml → 8 ml → RF = 13.5% → MR 0–1	59 ml → 8 ml → RF = 14.1% → MR 0–1	40 ± 24 ml	38 ± 24 ml
EROA (cm^2^)	0.31 ± 0.10	0.31 ± 0.11	0.41 ± 0.15	0.40 ± 0.15	0.41 ± 0.15	0.40 ± 0.15	0.25 ± 0.15	0.24 ± 0.14

*BSA* body surface area, *CI* left ventricular cardiac index, *CO* left ventricular cardiac output, *EROA* effective regurgitant orifice area, *HR* heart rate, *LVEDV* left ventricular end-diastolic volume, *LVEF* left ventricular ejection fraction, *LVESV* left ventricular end-systolic volume, *LVSV*_*Dopp*_ left ventricular effective (= forward) stroke volume determined by Doppler echocardiography, *LVSV*_*eff*_ left ventricular effective (= forward) stroke volume, *LVSV*_*tot*_ left ventricular total stroke volume determined by left ventricular planimetry, *RegVol*_*MV*_ transmitral regurgitant volume

As mentioned above, CO and CI are represented by the multiplication of LVSV_eff_ × HR and not by the multiplication of LVSV_tot_ × HR. Describing the conditions of a LVEDV of 200 ml and a LVEF of 30% at a normal HR of 65/min, the periphery needs the complete 60 ml of the LVSV_tot_ as LVSV_eff_ to ensure sufficient normal CO or CI as illustrated by the red dot (Fig. 3). If moderate FMR with a regurgitant fraction (RF) of 33% is assumed, this left ventricle is characterized by a LVEDV of 300 ml at a LVEF of 30% or by a LVEDV of 200 ml at a LVEF of 45% (brightened red dot in Fig. 3). If severe FMR with a RF of 50% is assumed, this left ventricle is characterized by a LVEDV of 400 ml at a LVEF of 30% or by a LVEDV of 200 ml at a LVEF of 60% (brightened red dot in Fig. 3).

**Fig. 4:**
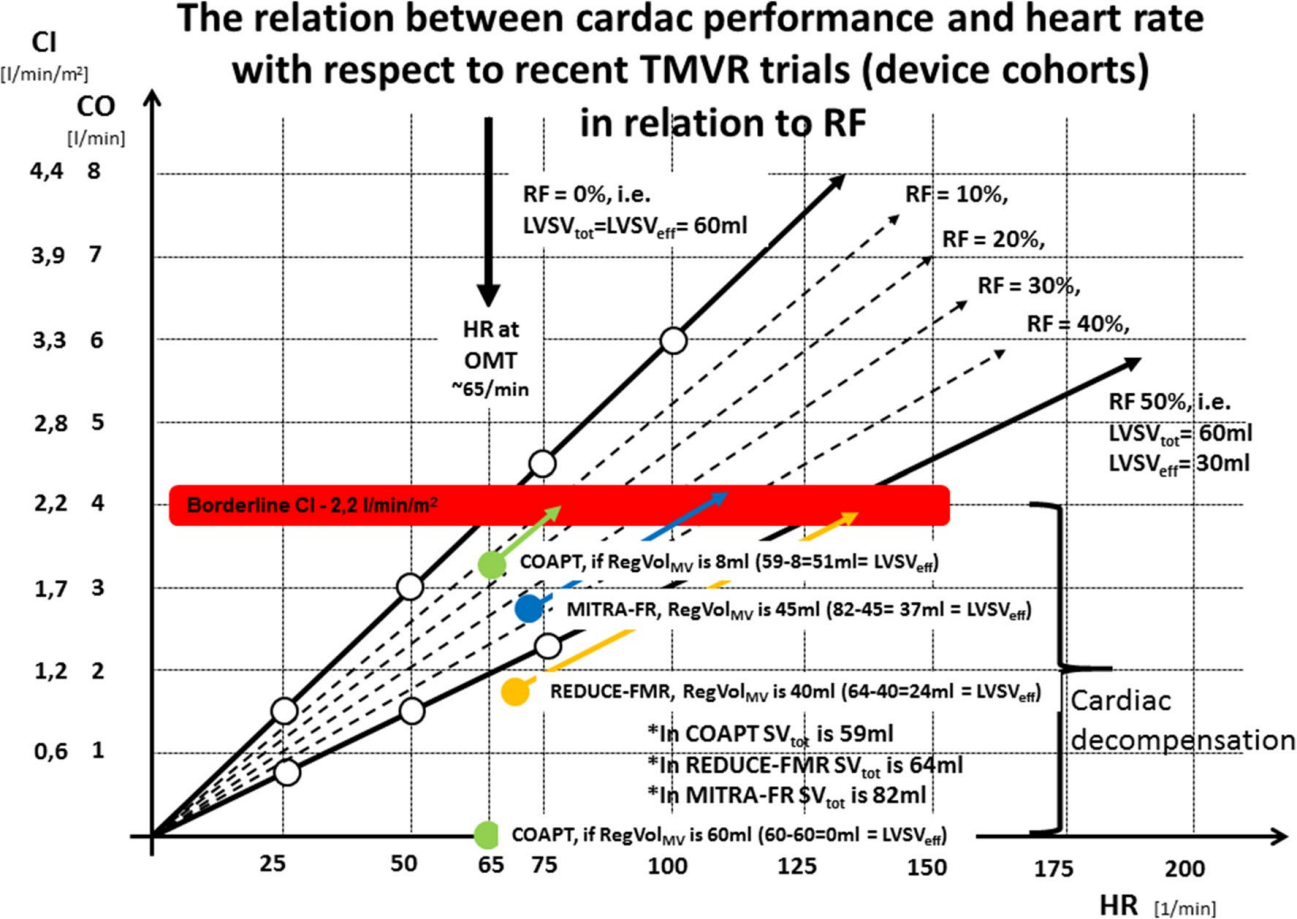
The relation between cardiac performance (*CO* cardiac output, *CI* cardiac index) and heart rate (HR) with respect to regurgitant fraction (RF): *LVSV*_*tot*_ total left ventricular (LV) stroke volume, *LVSV*_*eff*_ effective LV stroke volume, *RegVol*_*MV*_ regurgitant volume, *OMT* optimal medical treatment. Mean values of CI and HR presented in recent transcatheter mitral valve repair (TMVR) trials are illustrated by colored dots (green dots COAPT-version 1: LVSV_tot_ = 59 ml, RegVol_MV_ 59 ml, HR presumably about 70/min during OMT; version 2-LVSV_tot_ = 59 ml, LVSV_eff_ = 51 ml, RegVol_MV_ 8 ml, HR presumably about 70/min during OMT), blue dots MITRA-FR (LVSV_tot_ = 82 ml, RegVol_MV_ 45 ml, HR 73/min), and orange dots REDUCE-FMR (LVSV_tot_ = 64 ml, RegVol_MV_ 40 ml, HR 70/min). Thus, calculated HR in the presence of the reported RF to ensure a CI > 2.2 l/min/m^2^ is illustrated by colored arrows (COAPT-version 1: not possible; version 2 about 77/min), MITRA-FR about 110/min, REDUCE-FMR about 130/min) (additional explanations in the text)

In a CI–HR diagram with respect to different RF a borderline region to differentiate between normal conditions and cardiac decompensation can be marked at the limit of CI of 2.2 l/min/m^2^ (red bar in Fig. 4). In accordance with the previous diagrams (Figs. 2, 3) LVSV_eff_ must be ≥ 60 ml and HR ≥ 65/min to ensure a CI within normal ranges. The slope of the CI–HR-relationship decreases with increasing RF (dotted lines in Fig. 4). The pathophysiological consequence to ensure a sufficient CO or CI is the increase of HR (red bar in Fig. 4).

If the mean values of CI and HR of the recent TMVR trials [1–3] are put into the diagram, the colored dots represent the respective relationships (Fig. 4). CI-values of the recent TMVR trials were taken or calculated by the LVSV_tot_, LVSV_eff_, and RegVol_MV_-values presented in the literature [1–3]. In COAPT HR was not listed, thus, a HR of 65–70/min for cardiovascular patients under OMT was taken for calculation. As obviously shown, all colored dots are below the red bar, which represents the borderline range of CI between normal conditions and cardiogenic shock. As also illustrated by the arrows HR has to be much higher in all recent TMVR trials than presented in the literature or then the HR ranges of OMT to ensure CI > 2.2 l/min/m^2^ (colored arrows in Fig. 4).

The well-known EROA–LVEDV diagram adapted according to Grayburn et al. [12] should illustrate the proportionality between EROA and LVEDV in patients with severe FMR with a border area. Below this area non severe FMR is characterized. Above this area Grayburn et al. proposed to use the term “disproportionate” severe FMR [12]. Grayburn et al. described the diagnostic scenario that “physicians should seek to determine whether the estimated degree of MR is expected or proportionate to the degree of LV dilatation, or alternatively, whether the severity of MR is unexpected or disproportionate to the degree of LV enlargement” [12].

**Fig. 5:**
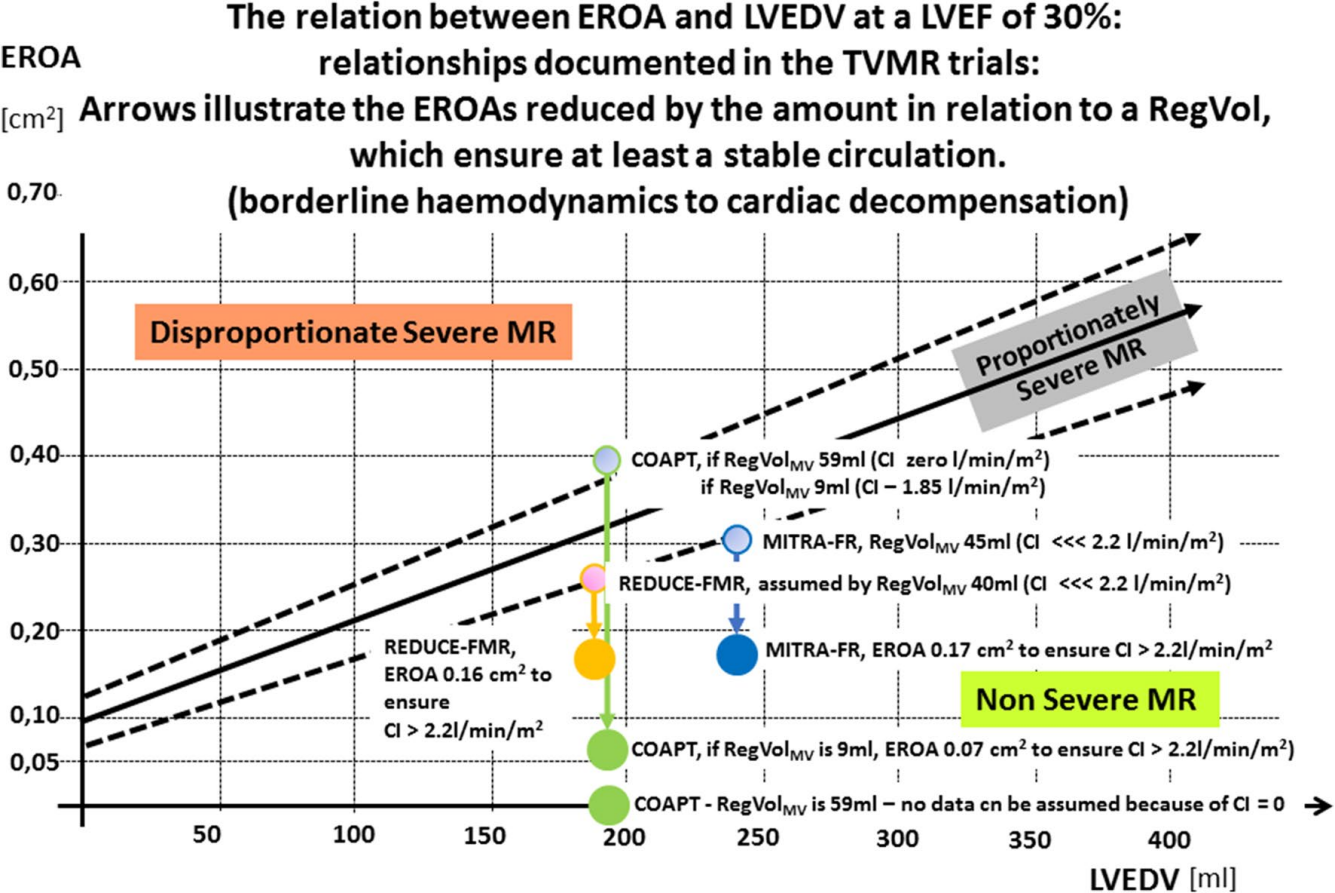
The relation between effective regurgitant orifice area (EROA) and left ventricular (LV) end-diastolic volume (LVEDV) at a LV ejection fraction (LVEF) about 30% [6]: the arrows illustrate the respective calculated EROAs presented in recent transcatheter mitral valve repair (TMVR) trials reduced by the amount in relation to a regurgitant volume (RegVol_MV_), which ensure at least a cardiac index (CI) > 2.2 l/min/m^2^. Mean values of EROA and LVEDV are given for green hollow dots COAPT [2, 10, 12], blue hollow dots MITRA-FR [1], and pink hollow dots REDUCE-FMR (assumed mean EROA of 0.26 cm^2^) [3]. If the EROA is reduced according to laws of rheology and physics, the EROAs of the filled circles have to be assumed in the recent TMVR trials (green dots COAPT, blue dots MITRA-FR, and orange dots REDUCE-FMR) (additional explanations in the text)

If the mean values of EROA and LVEDV of the recent TMVR trials [1–3] are put into the diagram, the brightened colored dots represent the respective relationships (Fig. 5). However, if these values are corrected with respect to plausible hemodynamics—that means RegVol_MV_ or EROA were reduced to ensure at least borderline CI of 2.2 l/min/m^2^—all these brightened dots shift into the area of non-severe FMR (colored dots in Fig. 5).

In all figures two dots are presented for the COAPT trial. The two dots are explained by the values reported in the literature about COAPT [2, 4]. The first reported LVSV_tot_ by planimetry in COAPT was 51 ml and the RegVol_MV_ was 59 ml which suggests overestimation of EROA and RegVol_MV_, underestimation of LVEDV, or both due to the impossibility of the specified values [2]. If 51 ml as LVSV_eff_ in the presence of 60 ml of LVSV_tot_ are assumed, a fully different scenario with a mild FMR of 8 ml with a corresponding RF of 14% is described (device cohort).

## Conclusions

With respect to hemodynamic implausibility of the echocardiographic data presented in the recent TMVR trials [1–3] it might be allowed to search for the reasons for this scenario. If RegVol_MV_, LVEDV, LVEF and LVSV_tot_ are not conclusive and plausible, the following conclusions can be drawn: RegVol_MV_ is obviously overestimated and planimetry-derived LVEDV is obviously underestimated. Thus, either the methods used should not be applied anymore or they should be applied correctly.

The assessment of MR severity by the echocardiographic “integrated approach” described by the current guideline recommendations is primarily based on semiquantitative analysis of semiquantitative parameters describing MR severity [8, 9, 23]. However, these parameters including the 2D-PISA approach have inherent problems to be methodically prone to errors. The colour flow jet area in the left atrium and its relation to the left atrial size depends on several methodological, anatomic, and pathophysiological factors [26]. Because of many factors, no standardization to adjust the colour flow jet area in MR patients is possible. In consequence, the approach of MR grading based on colour flow jet area is not recommended anymore [8]. The vena contracta (VC) by colour coded Doppler was described in MR patients in the parasternal long axis view because of better axial resolution in comparison to inferior lateral resolution in apical views [27]. Prerequisite of the VC method is the acquisition of the correct longitudinal sectional plane through the regurgitant jet to minimize underestimation beside methodological factors of ultrasound machine settings. Considering the mathematical model of the 2D-PISA method the frequent misuse can be explained e.g. by a inappropriate application in eccentrical jet formations or by an overestimation of RegVol_MV_ by measuring improper PISA radius at false time points and/or at the VC instead of the entry of the EROA [28]. The shape of the EROA and the jet direction are colour coded parameters yielding information to assume a relevant MR [9]. However, both entities should serve as a starting point for a quantitative MR assessment. The systolic flow reversal in the pulmonary veins is influenced by the jet direction towards the respective pulmonary veins, the size of the left atrium, and LV contractility causing over- as well as underestimation of MR severity [29]. The intensity of the MR-signal of the transmitral regurgitation using continuous wave Doppler is not recommended for assessing MR severity due to several practical and methodological limitations [8]. The ratio between transmitral velocity time integral (VTI) and flow velocity within the LVOT (VTI_MV_/VTI_LVOT_) seems to be suitable for a grading approach of MR severity, if the sample volumes are properly positioned, ultrasound beam alignment is parallel to the blood flow, MV stenosis or aortic valve regurgitation as well as mitral annular dilatation and atrial fibrillation is absent [30]. Due to all these multiple limitations of the semiquantitative parameters it might be necessary to assess quantitatively LVSV_tot_, LVSV_eff_ and RegVol_MV_ in a similar approach as currently used in cardiac magnetic resonance, CMR [31–33]. If echocardiographic measurements provide inconsistent results for LVSV_tot_, LVSV_eff_ and RegVol_MV_, the most likely explanation are obviously measurement errors due to methodological factors.

In HF patients with FMR recently two “hemodynamic pathways” have been described to characterize pathophysiological differences [19]. Firstly, LV remodeling defined by LV hypertrophy, LV dilatation, and LV sphericity causes papillary muscle displacement and widening of the mitral annulus with a consecutive FMR. This FMR type should be characterized by a linear relationship between LVEDV and EROA and has been named “proportionate MR”. Secondly, a “disproportionate FMR type” should be possible mainly due to LV dyssynchrony based on electrical conduction delay. This FMR type should be characterized by a greater MR severity than expected solely by LVEDV changes independent of LV geometry [19]. The percentage of HF patient with bundle branch block and FMR in the recent TMVR trials, who primarily are candidates for resynchronization therapy according to recent guidelines [34], has not been transparently presented. However, it was suggested that localized LV remodeling—especially regional wall motion abnormalities of the inferoposterior or lateral wall”—can induce dyssynchronous contraction resulting in a relevant “disproportionate FMR”.

However, the introduction of the terms “proportionate” and “disproportionate” FMR is misleading, because RegVol_MV_ has to be proportional to EROA in a system of communicating vessels (Fig. 1) at a single beat-to-beat measurement. Considering the single timepoint measurement of the PISA radius within the cardiac cycle there are sources of errors in quantifying RegVol_MV_ due to the dynamic nature of MR within systole. However, if RegVol_MV_ is under- or overestimated by this 2D-PISA approach, it is simply a measurement error, which reflects an incorrect assessment of RegVol_MV_. Inherently, physical laws of conservation of mass and energy cannot be neglected using echocardiography meaning that blood flow velocities at defined orifices are proportional in a system of communicating tubes (Fig. 1). Thus, the basic question raises whether a “disproportionate” FMR can be possible anyway. Instead of arithmetic juggling with inconsistent data the aim of a comprehensive echocardiography should be the correct assessment of conclusive values of LVSV_eff_, LVSV_tot_, LVEF and RegVol_MV_ in patients with FMR.

A complete other discussion are dynamic changes of the MR severity with changes of pre- and afterload [35]. It is obviously that RF is altered with increasing CO at the same heart rate, if preload or afterload is decreased, e.g. during sedation. It can be assumed, that RF will disproportionately increase with increasing afterload in FMR patients with advanced HF stages. To proof this concept of an “overproportionate FMR” at minimum two timepoint measurements at different afterload conditions are necessary. It can also be speculated that increased LV wall stress causes more LV remodeling with PM displacement, MV annulus dilatation and leaflet tenting supporting a higher risk of the development of relevant FMR described by the concept of “MR begets MR” in congestive HF [36]. However, again to proof this concept at minimum two timepoint measurements during follow-up under comparable circulatory conditions are necessary. The dynamic changes of MR severity with changes of pre- and afterload underline the importance to standardize measurement conditions during echocardiography—especially in TMVR trials. It is highly questionable to accept a baseline TTE within 90 days and a baseline TEE within 180 days prior to intervention as described in the COAPT Supplementary Appendix [2]. Baseline characteristics defined in this way might be scrutinized with respect to the possible changes of FMR severity due to several reasons. A comparison between a pre-interventional state the day before intervention and a post-interventional state at hospital discharge during comparable conditions—especially at the same heart rate, the same systemic blood pressure and the same drug treatment—should be the prerequisite for a verifiable documentation in clinical trials.

This unfortunate situation of implausible echocardiographic assessment in FMR patients mandates the integration of “hemodynamic conclusiveness” into the recent “integrated approach” [8, 9, 18]. New diagnostic algorithms apart from guidelines [8, 9] to „identify echocardiographic characteristics that predict favourable outcomes after TMVR in heart failure patients with severe secondary MR “ [10] are highly debatable—especially if inconsistent data are followed by treatment recommendations. A greater transparency of all trial data would presumably be helpful for a better understanding. The incongruencies of the reported hemodynamic values in patients with FMR in the recent TMVR trials illustrates the recent echocardiographic weakness in routine practice. The term “disproportionate” FMR is hardly to accept because a disproportionateness of hemodynamics might be just a proof of measuring error or simply a myth.

The objective, reproducible and transparent assessment of echocardiographic parameters for LV function and RF estimation in FMR patients will be the key for a proper decision making.

## Proposal of an extended transparent echocardiographic documentation focusing on hemodynamic plausibility in FMR patients FMR

**Fig. 6:**
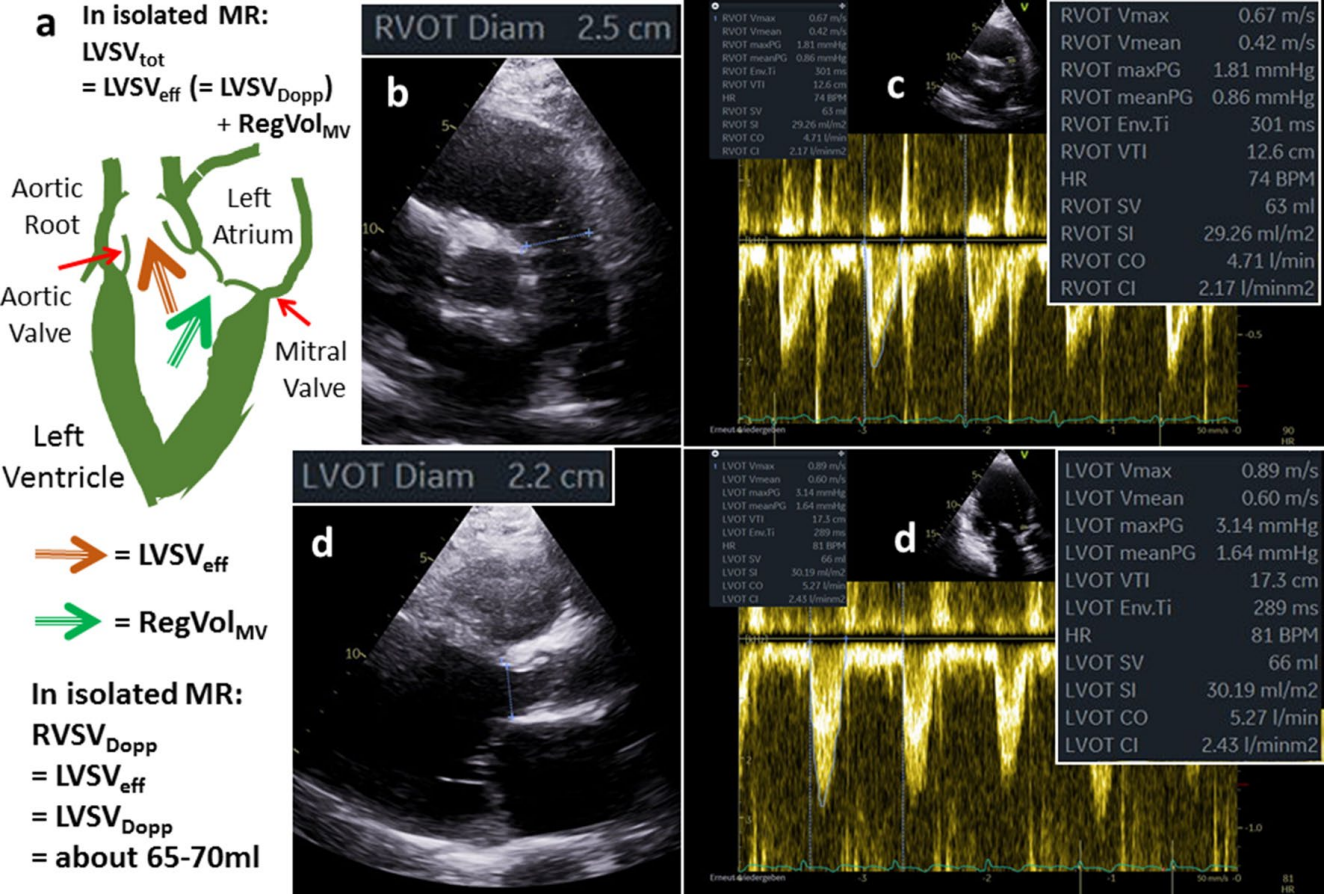
Illustration of quantitative assessment of left ventricular (LV) volumes in a patient with isolated functional mitral regurgitation (FMR)—part 1: a scheme of LV effective stroke volume (LVSV_eff_) and regurgitant volume (RegVol_MV_) in isolated FMR (**a**); measurements of right ventricular (RV) and LV stroke volume by Doppler echocardiography (RVSV_Dopp_, LVSV_Dopp_) by determination of the diameter of the RV outflow tract (RVOT) (**b**) and the velocity time integral (VTI) of the RVOT (RVOT VTI) (**c**) and by determination of the diameter of the LV outflow tract (LVOT) (**d**) and the LVOT VTI (). In isolated FMR RVSV_Dopp_ is equal to RVLV_Dopp_, which represents LVSV_eff_ as well as RVSV_eff_. LVSV_eff_ is between 65 and 70 ml in this case

**Fig. 7:**
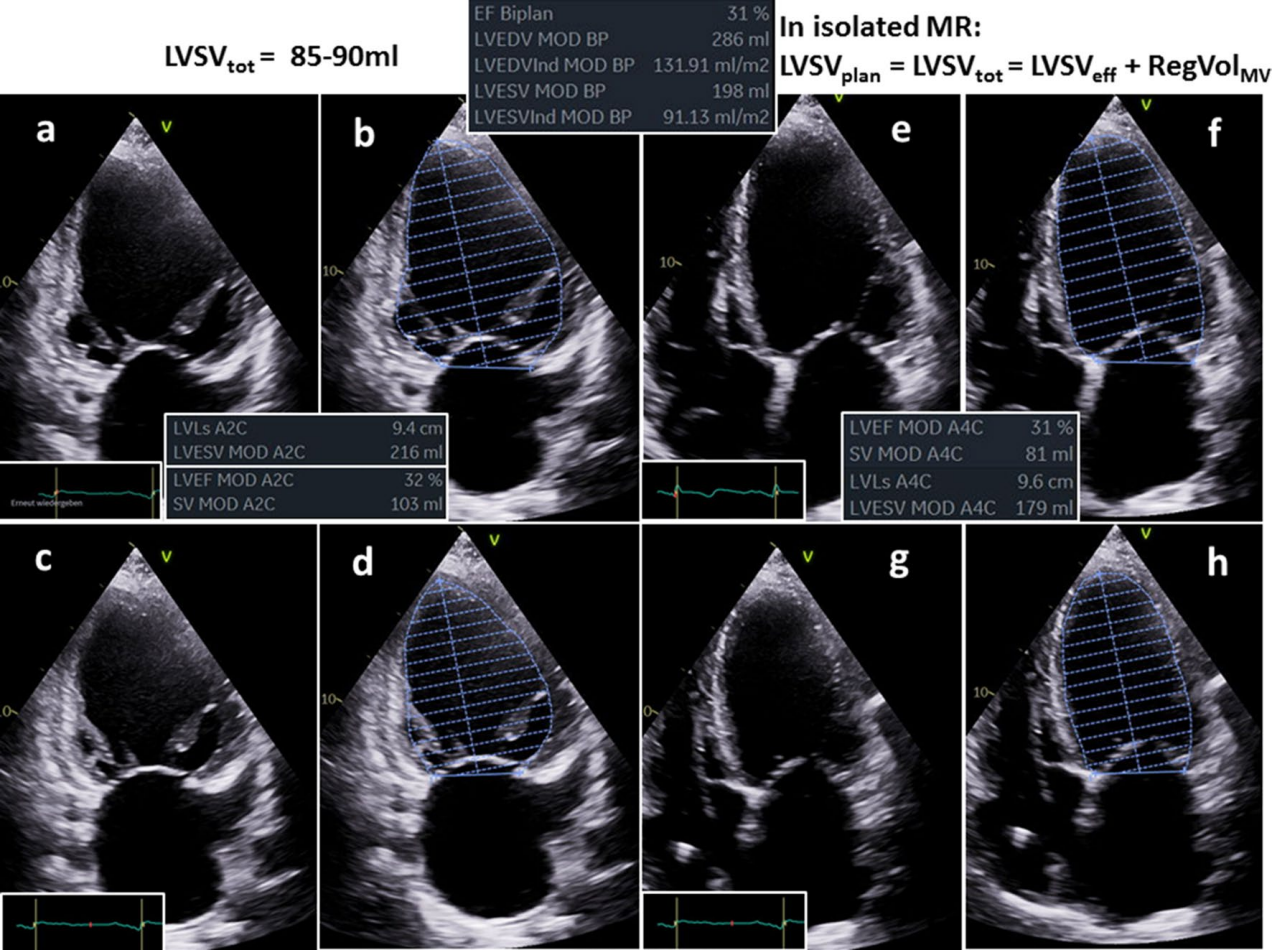
Illustration of quantitative assessment of left ventricular (LV) volumes in the same patient with isolated functional mitral regurgitation (FMR) as shown in Fig. 1—part 2: determination of left ventricular (LV) total stroke volume (LVSV_tot_) by biplane planimetry using Simpson’s method; planimetry of the 2-chamber view during diastole (**a**, **b**) and systole (**c**, **d**), planimetry of the 4-chamber view during diastole (**e**, **f**) and systole (**g**, **h**); the biplane LV planimetry enables the determination of LVSV_tot_, which is the sum of LV effective stroke volume (LVSV_eff_) and of the regurgitant volume (RegVol_MV_); LVSV_tot_ is between 85 and 90 ml in this case

**Fig. 8:**
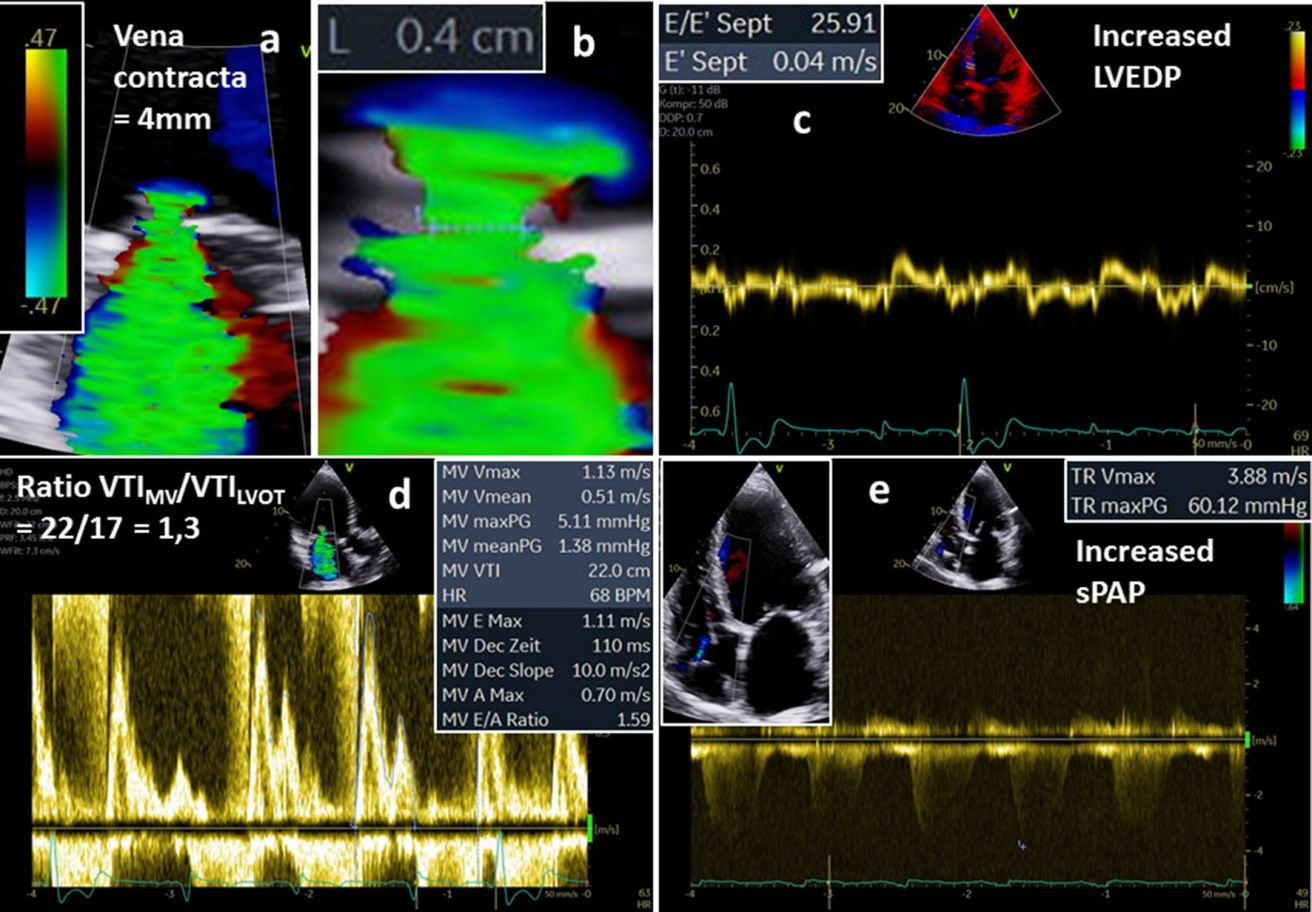
Illustration of quantitative assessment of left ventricular (LV) volumes in the same patient with isolated functional mitral regurgitation (FMR) as shown in Figs. 1 and 2—part 3: documentation of the FMR with a central jet formation, the jet area, the proximal convergence areas, and the vena contracta (VC) with a Nyquist limit of 47 cm/s (**a**), VC measurement with 4 mm (**b**), the basal septal myocardial velocity for calculation of E/E′ (**c**), the pulsed wave Doppler spectrum for determination of E velocity, the E/A-ratio and the ratio between transmitral velocity time integral (VTI) and flow velocity within the LVOT (VTI_MV_/VTI_LVOT_) (**d**), and the continuous wave Doppler spectrum of the transtricuspid regurgitation for estimation of the systolic pulmonary arterial pressure (sPAP) (**e**); increased E/E′, increased E-velocity, increased VTI_MV_/VTI_LVOT_ and increased sPAP document the secondary cardiac alterations of a relevant MR

**Fig. 9:**
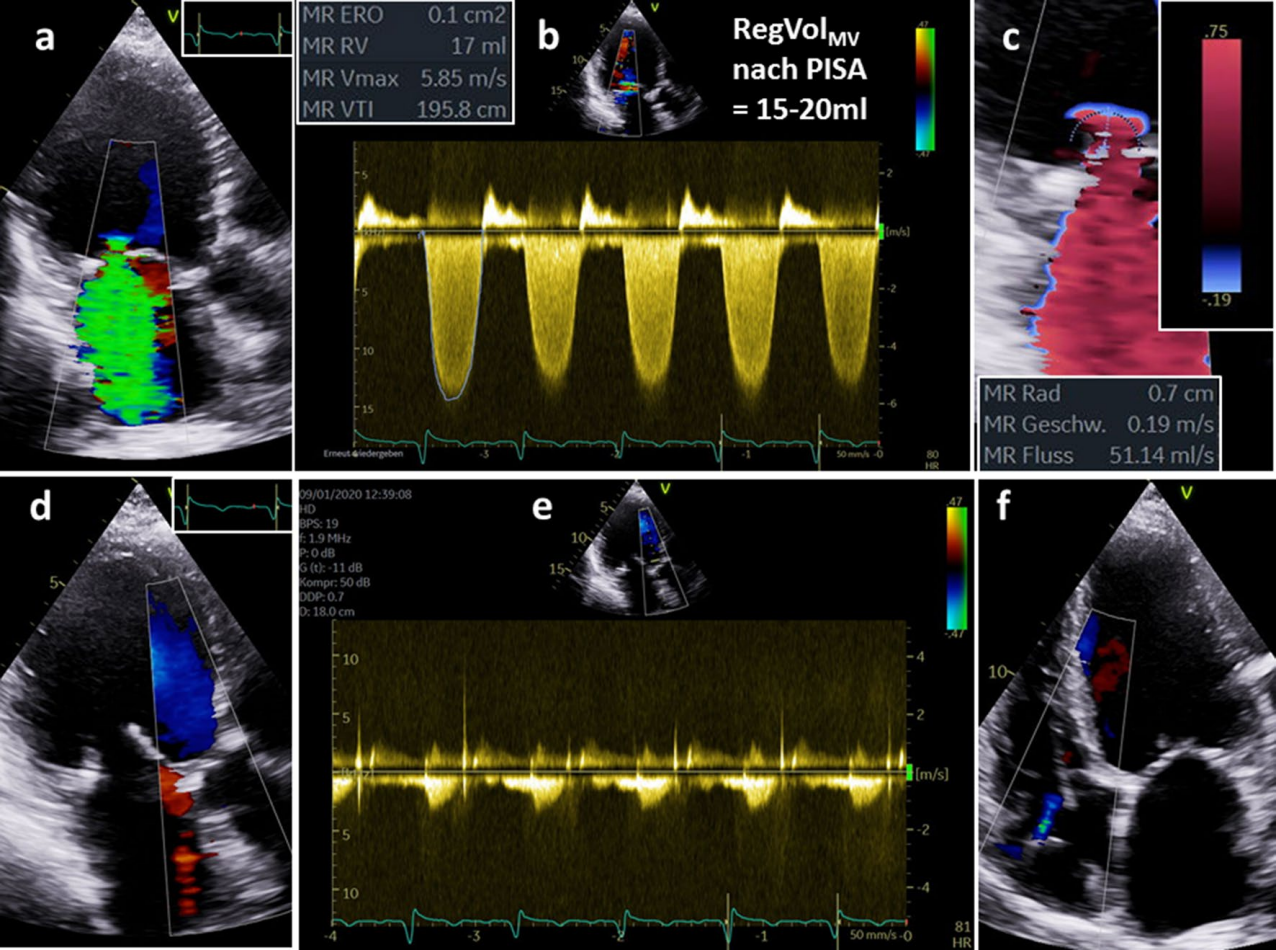
Illustration of quantitative assessment of left ventricular (LV) volumes in the same patient with isolated functional mitral regurgitation (FMR) as shown in Figs. 1, 2 and 3—part 3: determination of regurgitant volume (RegVol_MV_) by the 2D-PISA method—the jet phenomenon at systole using a Nyquist limit 47 cm/s (**a**), measurement of the mean and maximum regurgitant velocities (**b**), labeling of the 2D-PISA radius at a Nyquist limit of 19 cm/s (**c**), the estimation of RegVol_MV_ in this case, in which the 2D-PISA method is allowed to be used, results in a value of about 15–20 ml; the volumes LVSV_tot_, LVSV_eff_, RVSV_eff_, and RegVol_MV_ have to be conclusive and plausible; exclusion of additional relevant valvular diseases (**d**–**f**); color-coded image of the left ventricular outflow tract during diastolic documents the exclusion of aortic regurgitation (AR) (**d**), the respective continuous wave Doppler spectrum documents no regurgitant signal (**e**), and a trace tricuspid regurgitation during systole is documented (**f**)

A comprehensive echocardiography should integrate the estimating of cardiovascular parameters by a plausibility-check of the data. Despite the fact, that all cardiologists know, that the accurate assessment of LV volumes and LV function by echocardiography is well possible and methodological limitations in measuring LV volumes and LVEV have to be considered, the echocardiographic documentation with respect to its transparency, reproducibility and objectivity is illustrated regarding to the intention of an imaging journal (Figs. 6, 7, 8, 9).

The quantitative approach of FMR assessment by echocardiography is challenging. The quantitative assessment of LV volumes is highly criticized because of the necessity to determine several parameters, which are all prone to measuring errors that are squared in the respective calculations [8, 9]. Nevertheless, in isolated FMR, the LVSV_tot_ is determined by LV planimetry using the monoplane, biplane, triplane or 3D approach. The LVSV_eff_ is measured by Doppler calculations of forward stroke volume using cross sectional area of the LVOT and the PW-Doppler velocity time integral (VTI) of the LVOT. In patients with combined aortic valve disease the LVSV_eff_ can be determined by Doppler calculations of SV using cross sectional area the right ventricular outflow tract (RVOT) and the respective PW-Doppler VTI of the RVOT which is not common use in clinical routine echocardiography. As illustrated LV volumes and RegVol_MV_ can be correctly calculated by the differences of LVSV_tot_ and LVSV_eff_ or RVSV_eff_, and in few cases, in which the 2D-PISA method can adequately be used, the calculated RegVol_MV_ corresponds to the RegVol_MV_ determined by the 2D-PISA method (Figs. 6, 7, 8, 9). If all parameters can be assessed, a cross-check can be well performed with respect to plausible hemodynamics [37]. To provide a precise echocardiographic characterization of FMR severity the presented quantitative TTE approach might be additionally added to the “up to now integrated approach” to provide a more reliable and more consistent characterization of the FMR severity.

## Summary

1. The inconsistencies of the echocardiographic characterization of FMR severity make interpretations about FMR characteristics or generation of algorithms based on the trial results difficult.
2. The term “disproportionate FMR” is not in line with the physical laws of conservation of mass and energy and can only be explained by inconsistent echocardiographic data. Thus, the term is confusing and should therefore be avoided because a “disproportionate FMR” with inconsistent hemodynamics is not possible anyway.
3. A quantitative approach of FMR grading that includes the accurate quantitative assessment of LVSV_tot_, LVSV_eff_, RegVol_MV_ and individual RF by echocardiography should be discussed in future recommendations.
